# Risk Factors for Predicting Lymph Nodes Posterior to Right Recurrent Laryngeal Nerve (LN-prRLN) Metastasis in Thyroid Papillary Carcinoma: A Meta-Analysis

**DOI:** 10.1155/2019/7064328

**Published:** 2019-03-31

**Authors:** Cunfu Li, Jun Xiang, Yunjun Wang

**Affiliations:** ^1^Department of Thyroid Surgery, Weihai Central Hospital, Weihai, China; ^2^Department of Head and Neck Surgery, Fudan University Shanghai Cancer Center, Shanghai, China; ^3^Department of Oncology, Shanghai Medical College, Fudan University, Shanghai, China

## Abstract

**Objective:**

To evaluate the risk factors for predicting lymph nodes (LN) posterior to right recurrent laryngeal nerve metastasis in thyroid papillary carcinoma.

**Methods:**

PubMed, PMC, EMBASE, and the Cochrane Library were systematically searched for articles published spanning 30/06/2009-30/8/2018 using multiple search terms. Thirteen articles involving 10,014 patients were reviewed in our meta-analysis. Stata 15.1 software was used for the meta-analysis.

**Results:**

The rate of LN posterior to right recurrent laryngeal nerve (LN-prRLN) metastasis was 8.65%. Univariate analysis showed that age (*P* = 0.001), gender (*P* < 0.001), tumour size (*P* < 0.001), lateral LN metastasis (*P* < 0.001), extrathyroidal invasion (*P* < 0.001), multifocality (*P* = 0.005), capsule invasion (*P* < 0.001), tumour location (*P* = 0.076), lymph nodes anterior to right recurrent laryngeal nerve (LN-arRLN) metastasis (*P* < 0.001), and central LN metastasis (*P* < 0.001) were significantly associated with the increased incidence of LN-prRLN metastasis in thyroid papillary carcinoma.

**Conclusion:**

PTC patients aged <45, male, and with tumours > 1 cm, lateral LN metastasis, extrathyroidal invasion, multifocality, capsule invasion, LN-arRLN metastasis, or central LN metastasis were significantly correlated with lymph nodes posterior to right recurrent laryngeal nerve metastasis, indicating LN-prRLN dissection.

## 1. Introduction

According to the World Health Organization classification system for thyroid carcinoma, papillary thyroid carcinoma (PTC) is the most common endocrine cancer, with increased mortality [1, 2]. PTC accounts for 80% of thyroid cancers and is one of the fastest-growing cancers among cancer patients [3]. It has an excellent prognosis, with a 10-year survival rate exceeding 90% and a 15-year survival rate exceeding 88%. American Thyroid Association (ATA), National Comprehensive Cancer Network (NCCN), and AJCC (American Joint Committee on Cancer) recommend performing prophylactic central lymph node dissection. The central lymph nodes are subdivided into Delphian lymph nodes, paratracheal lymph nodes, and pretracheal lymph nodes. The right paratracheal lymph nodes are obviously different from the left based on anatomical differences. The right paratracheal lymph nodes can be divided into two parts by the right recurrent laryngeal nerve [4], and we refer to them as lymph nodes posterior to right recurrent laryngeal nerve lymph nodes (LN-prRLN) and lymph nodes anterior to right recurrent laryngeal nerve lymph nodes (LN-arRLN). In some reports, the LN-prRLN are also called right para-oesophageal lymph nodes (RPELNs) or the VIb compartment, and the LN-arRLN are called the VIa compartment. The proper range of the LN-prRLN is still controversial [5, 6]. As we know, performing entire right CLND dissection is more difficult for many thyroid surgeons for anatomical reasons. Because lymph nodes posterior to the right recurrent laryngeal nerve are attached to the recurrent laryngeal nerve, it is necessary to dissociate the right recurrent laryngeal nerve completely when we perform right CLND dissection. In general, complete right central lymph node dissection requires removal of the LN-arRLN and LN-prRLN compartments together. However, LN-prRLN dissection has not been performed completely for some time for three reasons: (1) increasing the risk of RLN injury, (2) complicated anatomy, and (3) excellent prognosis. Whether resection of the central lymph nodes is necessary for every patient is still controversial. Thus, many surgeons have ignored the lymph nodes posterior to right recurrent laryngeal nerve because of these complications. The approach of not clearing the LN-prRLN in all patients is not appropriate; therefore, we must identify some risk factors for predicting lymph nodes posterior to right recurrent laryngeal nerve metastasis in thyroid papillary carcinoma. Identifying the risk factors associated with LN-prRLN metastasis in PTC would assist surgeons in making decisions about whether to perform LN-prRLN dissection. To solve this problem, we conducted a meta-analysis to evaluate the related factors influencing PTC from the current literature.

## 2. Materials and Methods

### 2.1. Study Inclusion/Exclusion Criteria

This meta-analysis is reported in accordance with the Preferred Reporting Items for Systematic Reviews and Meta-Analyses (PRISMA) Statement and was registered with the International Prospective Register of Systematic Reviews (number CRD42018104820).

All articles identified by the search strategy were screened by two authors (CunFu Li, YunJun Wang). All studies were required to meet the following criteria for this study: (1) the diagnosis was PTC; (2) the study had a clear outcome with a definite pathological diagnosis; (3) all patients received total thyroidectomy or right lobectomy, and their right central lymph node must have been completely removed; (4) the location of the primary tumour was not limited; (5) the study language was not limited; and (6) the study was retrospective or prospective. We excluded studies that met at least one of the following criteria: (1) patients with mixed-type PTC; (2) patients were not receiving their initial treatment; (3) the works were comments, reviews, letters, expert opinions, meetings, or case reports; (4) it was impossible to extract the clear outcomes from the article; and (5) the article was a duplicate published by the same author.

### 2.2. Search Strategy

A comprehensive review of studies using the PubMed, EMBASE, and Cochrane databases for articles published from June 30, 2009, to August 30, 2018, was performed. We included only original peer-reviewed research reports of risk factors for lymph nodes posterior to right recurrent laryngeal nerve metastasis in thyroid papillary carcinoma. The following free-text search terms in “All fields” “Thyroid Neoplasms”[Mesh] (Neoplasm, Thyroid) OR (Thyroid Neoplasm) OR (Neoplasms, Thyroid) OR (Thyroid Carcinoma) OR (Carcinoma, Thyroid) OR (Carcinomas, Thyroid) OR (Thyroid Carcinomas) OR (Cancer of Thyroid) OR (Thyroid Cancers) OR (Thyroid Cancer) OR (Cancer, Thyroid) OR (Cancers, Thyroid) OR (Cancer of the Thyroid) OR (Thyroid Adenoma) OR (Adenoma, Thyroid) OR (Adenomas, Thyroid) OR (Thyroid Adenomas) “Lymphatic Metastasis”[Mesh] OR (Lymphatic Metastases) OR (Metastases, Lymphatic) OR (Metastasis, Lymphatic) “Recurrent Laryngeal Nerve”[Mesh] OR (Laryngeal Nerve, Recurrent) OR (Laryngeal Nerves, Recurrent) OR (Nerve, Recurrent Laryngeal) OR (Nerves, Recurrent Laryngeal) OR (Recurrent Laryngeal Nerves) OR (Laryngeal Nerve, Inferior) OR (Inferior Laryngeal Nerve) OR (Inferior Laryngeal Nerves) OR (Laryngeal Nerves, Inferior) OR (Nerve, Inferior Laryngeal) OR (Nerves, Inferior Laryngeal) OR “Paraoesophageal” OR “para-oesophageal” “Risk Factors”[Mesh] OR (Factor, Risk) OR (Factors, Risk) OR (Risk Factor) OR (Population at Risk) OR (Risk, Population at) OR (Populations at Risk) OR (Risk, Populations at) were used. There was no language restriction and no methodological filters. The search strategy was slightly adjusted according to the requirements of different databases. Review articles and bibliographies of other relevant identified investigations were hand-searched to identify additional studies.

### 2.3. Selection of Studies

The articles were screened manually for relevance by two independent investigators (Cunfu Li and Yunjun Wang). Any disagreement about including or excluding a study was resolved by discussing with SSK.

### 2.4. Data Extraction and Management

Two reviewers (Cunfu Li and Yunjun Wang) read full texts of potential articles and independently abstracted outcome data. Disagreements were solved by the third reviewer (Jun Xiang).

The following data were extracted from all the included articles: first author, country, years of publication, age, gender, tumour size, extrathyroidal extension, multifocality, capsule invasion, tumour location lateral LN metastasis, LN-arRLN metastasis, and central LN metastasis.

### 2.5. Statistical Analysis

Data were integrated using Stata 15.1 (Stata Corporation, College Station, TX, USA). For dichotomous variables, overall estimates of odds ratios (ORs) and 95% confidence intervals (CIs) were pooled using the random effect model.

Between-study heterogeneity was assessed using the *I*^2^ statistic. If 25%<*I*^2^ ≤ 50%, 50%<*I*^2^ ≤ 75%, and 75%<*I*^2^, we classified the heterogeneity as low, moderate, or high risk. For assessing the influence of each study on the overall effect size, we used the leave-one-out method to find the heterogeneity.

### 2.6. Risk of Bias

**Figure 1:**
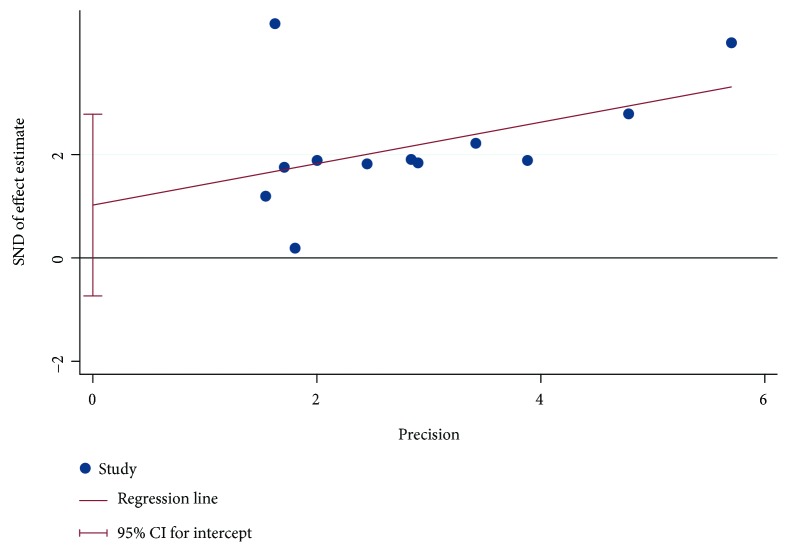
Risk of bias.

As the topic involves surgical procedures and outcomes, it is very likely that smaller studies or those with unfavourable outcomes may not be published in the literature. Thus, we used Egger's regression test and visual funnel plots to assess the presence of publication bias through the software Stata 15.1 (Stata Corporation, College Station, TX, USA). Funnel plots indicated no clear evidence of existing significant publication bias (*P* = 0.224) (Figure 1).

## 3. Results

We had 308 studies for title and abstract screening. After reading the title and abstract, 23 studies met our criteria and were chosen for full-text screening. After the full-text screening step, we excluded 10 articles, and 13 articles remained for analysis. The study characteristics between the 13 eligible studies are compared in Table 1.

### 3.1. Age of Patients

**Figure 2:**
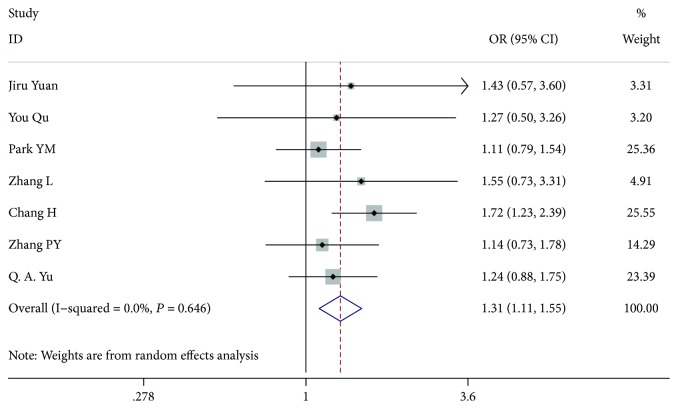
Forest plot for metastasis according to age.

Seven studies [7–12] included 3907 patients aged <45 years with PTC and 4507 aged ≥45 years with PTC (OR 1.314, 95% CI 1.111-1.554, *I*^2^ = 0.0%, *P* = 0.001). There was a significant difference between the 2 variants (Figure 2).

### 3.2. Gender

**Figure 3:**
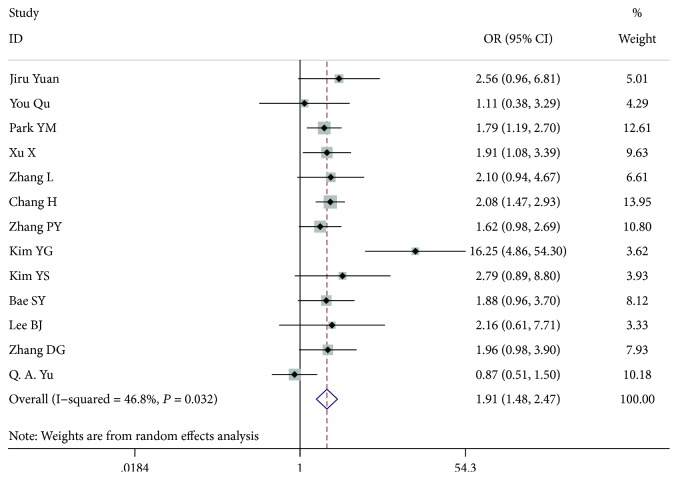
Forest plot for metastasis according to gender.

Thirteen studies [7–19], comprising 11,104 patients, 1988 males and 8026 females, were included for analysis. In the gender analysis, compared with the women, the men had a higher risk of LN-prRLN metastasis (OR = 1.913, 95% CI 1.481-2.471, *P* < 0.001, *I*^2^ = 46.8%) (Figure 3).

### 3.3. Tumour Size

**Figure 4:**
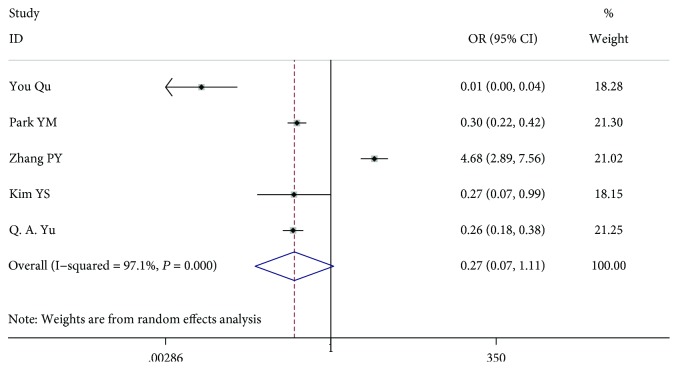
Forest plot for metastasis according to tumour size < 1 cm vs. tumour size ≥ 1 cm.

**Figure 5:**
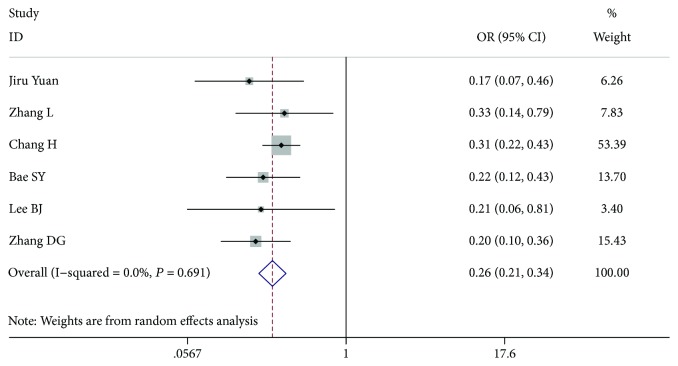
Forest plot for metastasis according to tumour size ≤ 1 cm vs. tumour size > 1 cm.

Eleven studies [7–13, 15–18] including 9541 patients were analysed. Because some articles (six articles) [7, 10, 11, 16–18] analysed tumour diameters > 1 cm and some articles (five articles) [8, 9, 12, 13, 15] analysed tumour diameters ≥ 1 cm, we divided the information into two groups and analysed the data. In the patient study analysis < 1 cm versus (vs.) ≥1 cm (OR = 0.275, 95% CI 0.068-1.106, *P* = 0.069, *I*^2^ = 97.1%), there was no significant difference between the 2 variants, and the study had a high heterogeneity. However, in the patient study analysis ≤ 1 cm vs. >1 cm (OR = 0.264, 95% CI 0.207-0.338, *P* < 0.001, *I*^2^ = 0.00%), there was a significant difference between the 2 variants, and the study had a low heterogeneity (Figures 4 and 5).

### 3.4. Multifocality

**Figure 6:**
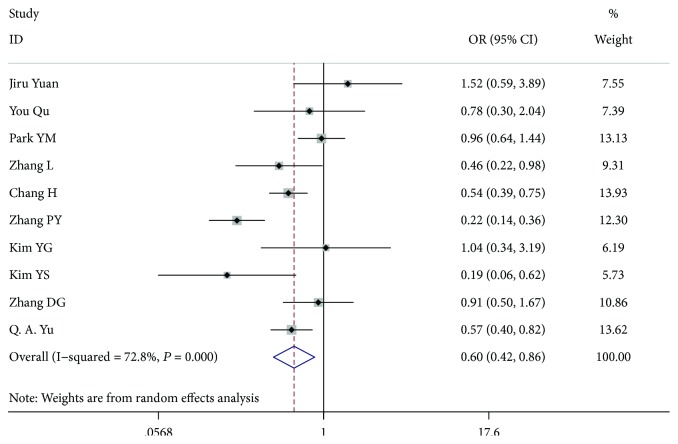
Forest plot for metastasis according to multifocality.

In total, 9134 patients from 10 studies [7–15, 18] were included for analysis. In the multifocality analysis, compared with no multifocality, the patients with multifocality had a higher risk of LN-prRLN metastasis (OR = 0.599, 95% CI 0.419-0.856, *P* = 0.005, *I*^2^ = 72.8%). After we excluded the article by Pinyi et al. [13], the among-study heterogeneity was not present (*I*^2^ = 47.9%), and the overall effect remained significant (OR = 0.686, 95% CI 0.518-0.908, *P* = 0.009) (Figure 6).

### 3.5. Capsule Invasion

**Figure 7:**
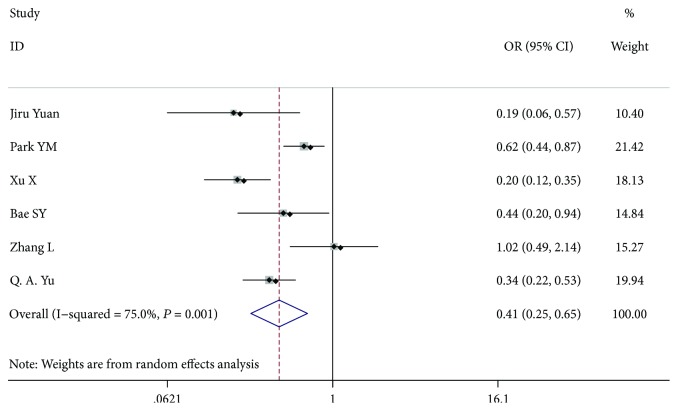
Forest plot for metastasis according to capsule invasion.

Six studies [7, 9, 10, 12, 16, 19] with 3040 patients, 1182 patients with capsule invasion and 1858 patients without capsule invasion, were included for analysis. The outcome showed that capsule invasion was a risk factor of LN-prRLN metastasis (OR = 0.407, 95% CI 0.253-0.655, *P* < 0.001, *I*^2^ = 75.0%). After we excluded the article by Xu et al., the among-study heterogeneity was not present, and the overall effect had a significant difference (OR = 0.481, 95% CI 0.309-0.750, *P* = 0.001, *I*^2^ = 63.5%) (Figure 7).

### 3.6. Extrathyroidal Invasion

**Figure 8:**
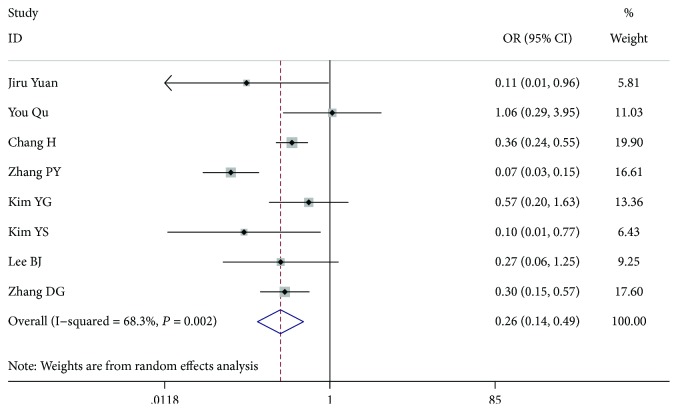
Forest plot for metastasis according to extrathyroidal invasion.

Eight studies [7, 8, 11, 13–15, 17, 18] including 7066 patients examined extrathyroidal invasion (3178 patients who were extrathyroidal-negative and 3888 patients who were extrathyroidal-positive) (OR = 0.264, 95% CI 0.142-0.491, *P* < 0.001, *I*^2^ = 68.3%). After we excluded the article by Pinyi et al. [13], the among-study heterogeneity was not present, and the whole study had a significant difference (OR = 0.357, 95% CI 0.251-0.506, *P* < 0.001, *I*^2^ = 7.8%) (Figure 8).

### 3.7. Tumour Location

Eight studies [7, 8, 10, 13–15, 18] including 1303 patients assessed tumour location. According to the vertical axis, the thyroid gland is divided into three equal parts: upper, middle, and lower. Because the analysis had three variables, we first performed an analysis between the upper thyroid gland and the middle thyroid grand. The patients with upper gland involvement had no significant statistics (OR = 1.710, 95% CI 0.945-3.094, *P* = 0.076, *I*^2^ = 49.2%). Then, we analysed the upper thyroid gland and the lower thyroid gland and found the same outcome: the upper gland had a higher risk of LN-prRLN metastasis (OR = 1.778, 95% CI 1.144-2.762, *P* = 0.010, *I*^2^ = 0.0%). Finally, we analysed the middle gland and the lower gland and found that patients with middle gland involvement had no significant statistics (OR = 0.867, 95% CI 0.611-1.231, *P* = 0.425, *I*^2^ = 0.0%).

### 3.8. Lateral LN Metastasis

**Figure 9:**
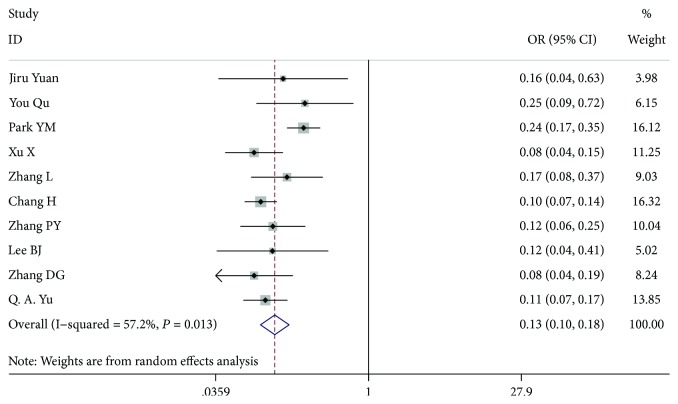
Forest plot for metastasis according to lateral LN metastasis.

Ten studies [7–13, 17–19], comprising 7904 patients who were lateral LN metastasis-negative and 1403 patients who were lateral LN metastasis-positive, were analysed, and there was a significant difference between them (OR = 0.130, 95% CI 0.095-0.178, *P* < 0.001, *I*^2^ = 57.2%). After we excluded the article by Park et al. [9], the among-study heterogeneity was not present, and the overall effect remained significant (OR = 0.108, 95% CI 0.087-0.133, *P* < 0.001, *I*^2^ = 0.0%) (Figure 9).

### 3.9. LN-arRLN Metastasis

**Figure 10:**
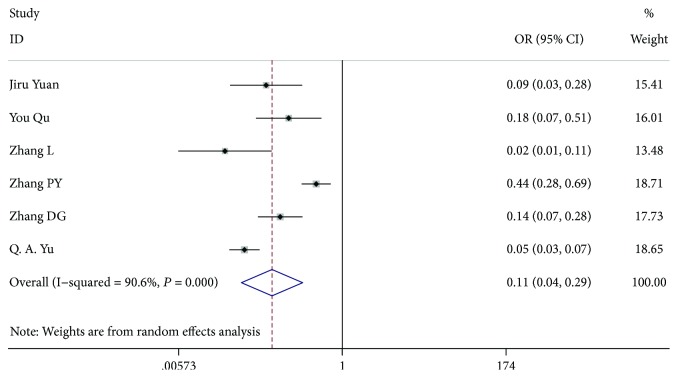
Forest plot for metastasis according to LN-arRLN metastasis.

Six studies [7, 8, 10, 12, 13, 18], comprising 2113 patients with LN-arRLN dissection, included 1339 patients who were LN-arRLN metastasis-negative and 774 patients who were LN-arRLN metastasis-positive. Patients with LN-arRLN metastasis were present in 39.6% (307 of 774), and patients without LN-arRLN metastasis were present in 7.09% (95 of 1339). There was a significant difference between the groups (OR = 0.109, 95% CI 0.041-0.286, *P* < 0.001, *I*^2^ = 90.6%). Because of the high heterogeneity, we excluded the article by Pinyi et al. [13]. Our study found that the among-study heterogeneity was lower and that the overall effect remained significant (OR = 0.080, 95% CI 0.040-0.160, *P* < 0.001, *I*^2^ = 68.7%) (Figure 10).

### 3.10. Central LN Metastasis

**Figure 11:**
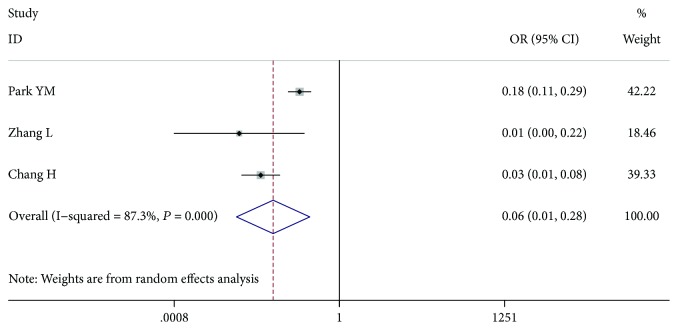
Forest plot for metastasis according to central LN.

Three studies [9–11], comprising 6909 patients with central LN dissection, included 3354 patients with central LN metastasis and 3555 patients without central LN metastasis. The patients with central LN metastasis had a higher risk of LN-prRLN metastasis (OR = 0.057, 95% CI 0.012-0.279, *P* < 0.001, *I*^2^ = 87.3%). After we excluded the article by Park et al. [9], the among-study heterogeneity was not present, and the overall effect remained significant (OR = 0.031, 95% CI 0.014-0.068, *P* < 0.001, *I*^2^ = 0.0%) (Figure 11).

## 4. Discussion

This study was the largest sample size meta-analysis of the literature exploring the factors for predicting lymph nodes posterior to right recurrent laryngeal nerve (LN-prRLN) metastasis in thyroid papillary carcinoma by searching for articles. At present, the name of the right central region of the posterior to the recurrent laryngeal nerve is still controversial. Some authors call this area the “lymph nodes posterior to the right recurrent laryngeal nerve [7, 13, 18, 19],” and some call the area the “right paraesophageal lymph nodes [8–12, 14–17].” The authors of this study thought that the name “lymph nodes posterior to the right recurrent laryngeal nerve” was more appropriate because the right recurrent laryngeal nerve must be sufficiently dissociated to expose the targeted lymph nodes.

Lymph nodes posterior to right recurrent laryngeal nerve (LN-prRLN) metastasis were not common and were present in 2.66%-26.67% of patients with PTC [7–19]. In our study, out of all patients, 8.65% (867 of 10014) had LN-prRLN metastases and underwent therapeutic or CLND dissection 8.65% (867 of 10,014). Because there has been much debate about lymph node metastases and the related impact on survival in PTC patients, CLND and prophylactic CLND remain controversial [20–22]. Some studies supported the idea that CLND may reduce recurrence and prolong survival [23–25]. However, many surgeons do not perform complete surgical resection in a CLND for reducing complications and shortening the operation time. Most patients do not relapse, and the prognosis was satisfied, but a few patients with regional recurrence in LN-prRLN metastasis were relatively more difficult to be reoperated upon and would encounter a higher possibility of complications [26], and the subsequent surgery needed to be performed by experienced surgeons [10]. LN-prRLN metastasis may result in a devastating clinical course by directly invading important organs and may lead to serious consequences. Therefore, we attempted to identify the risk factors for predicting lymph nodes posterior to right recurrent laryngeal nerve (LN-prRLN) metastasis in PTC.

Today, the eighth edition of the AJCC guide raises the age limit to 55 years, but most of our studies limited the age to 45 years old; thus, the age limit of 45 years was analysed. In some studies, age did not have a significant statistical difference in LN-prRLN metastasis [7–10, 12–19]. However, in our study, we found that age < 45 is a risk factor for right recurrent laryngeal nerve (LN-prRLN) metastasis in PTC. This finding may be due to an expansion of the sample size to make the results more meaningful. Eleven studies [7–10, 12, 14–18] reported that there was no significant statistical difference between male patients and female patients. After data combination, we found a significant difference between male patients and female patients, with the male patients having a higher risk of LN-prRLN metastasis than female patients.

As we know, multiplicity has been shown to be a dependent risk factor for predicting LN-prRLN metastasis in PTC. In our study, this result was proved again. Extrathyroidal extension is also known to be an adverse prognostic factor [27]. We previously proved that significant extrathyroidal extension was a strong predictor of LN-prRLN metastases in PTC. Some studies have shown that patients with larger tumour sizes were more likely to experience metastasis than patients with smaller tumours [7–13, 15–19]. Tumour size was a strong risk factor of LN-prRLN metastases in PTC [7–13, 15–18]. Tumour size has always been closely related to thyroid lymph node metastases. We selected tumours with diameters of 1 cm as the limit so that lymph node dissection could be strictly performed. Thirteen studies were analysed, and there were essentially no significant differences in the upper, middle, and lower locations. After data combination, we found that the upper tumour location had no significant difference compared with the middle tumour location; however, the upper tumour location had a higher risk of LN-prRLN metastasis compared with the lower location. This result may be associated with the fact that the location of the lymphatic drainage in the metastatic scope has closer ties than the lower location. Some studies reported that LN-arRLN metastasis was an independent predictor of LN-prRLN metastasis [10, 17]. In our study, the conclusion was proved again, and the central LN metastasis had the same outcome. Because of the characteristics of lymphatic drainage, when lateral LN metastasis was positive, LN-prRLN metastasis occurred easily. In our study, lateral LN metastasis was a risk factor of LN-prRLN metastasis. If there was a suspicious representation of LN-arRLN metastasis or lateral LN metastasis, it was quite necessary to perform LN-prRLN dissection.

In addition to the above indicators, Qu et al. [8] chose ultrasound suspect and CT-visible as indicators. According to this study, the specificity and sensitivity of ultrasound in evaluating LN-prRLN metastasis were 95.8% and 11.1%, respectively. The prevalence of LN-prRLN metastasis in the CT-visible group was significantly different compared with that in the CT-invisible group. However, only one study analysed the outcome, so the two indicators were not chosen. The use of ultrasound in detecting lymph node metastasis in LN-prRLN was limited, and the sensitivity of ultrasound for detecting metastatic nodes in the central compartment was 44.4% [10]. Regardless, the ultrasound indicator may be significant to LN-prRLN metastasis, and more data will be needed to prove it. In the future, ultrasound suspect must be the most important indicator for predicting LN-prRLN metastasis.

Two studies [8, 10] described an indicator as chronic lymphocytic thyroiditis, and there was no significant difference between them. Chronic lymphocytic thyroiditis was not found to be obviously associated with thyroid carcinoma. Therefore, chronic lymphocytic thyroiditis was not included in our study.

## 5. Conclusion

In conclusion, this study demonstrated that age < 45, male gender, tumours > 1 cmlateral LN metastasis, extrathyroidal invasion, multifocality, capsule invasion, LN-arRLN metastasis, and central LN metastasis were significant risk factors of lymph nodes posterior to right recurrent laryngeal nerve metastasis in patients with PTC and that we should perform LN-prRLN dissection in such patients.

## Figures and Tables

**Table 1:** Clinicopathological characteristics associated with metastasis to LN-rRLN in PTC patients.

Variables	Number of studies	LN-rRLN		*P*
		Metastasis (-)	Metastasis (+)	
Gender	11			<0.001
Male		1633	203	
Female		6632	454	
Age (y)	6			0.001
<45		3227	270	
≥45		3847	241	
Tumour location	8			0.076
Upper		220	60	
Middle		568	107	
Lower		341	82	
Tumour size (cm)	11			
≤1		4301	113	<0.001
>1		2143	207	
<1		988	156	0.069
≥1		701	248	
Multifocality	10			0.005
Yes		2759	253	
No		5038	355	
Capsular invasion	5			<0.001
Yes				
No				
Extrathyroidal invasion	9			<0.001
Yes		3687	224	
No		3106	194	
Lateral LN metastasis	9			<0.001
Yes		1022	242	
No		6603	333	
VIa central LN metastasis	4			<0.001
Yes		334	171	
No		709	70	
Central LN metastasis	5			<0.001
Yes		3099	340	
No		3587	28	

## Data Availability

The data used to support the findings of this study are included within the article.
